# Comment on “Retained Placenta Accreta Mimicking Choriocarcinoma”

**DOI:** 10.1155/2016/9819584

**Published:** 2016-01-03

**Authors:** Mehmet Aral Atalay, Bilge Cetinkaya Demir

**Affiliations:** Department of Obstetrics and Gynecology, Uludağ University, 16059 Bursa, Turkey

We read the presented case of retained invasive placenta mimicked gestational choriocarcinoma (GCC) with an enthusiasm [1]. We thought if this is a case of GCC, which sign should be the leading sign.

The clinical diagnosis of GCC is challenging in most of the cases. The predominant symptom is abnormal vaginal bleeding. Serum human chorionic gonadotropin beta (*β*-hCG) measurement and doppler ultrasonography examination are the leading diagnostic work-ups. Contrast-enhanced MRI is also useful for detecting an abundant blood flow in the tumor.

In this case, authors did not consider to use MRI study. The authors demonstrated a serum *β*-hCG level of 203 IU/L, which is unlikely in cases with GCC. One should expect *β*-hCG measurements exceeding 100.000 IU/L in GCC [2]. This finding decrease its likelihood to be a GCC. Instead of a GCC, authors could compose their theory on the other types of gestational trophoblastic neoplasia, which are placental site trophoblastic tumor, epithelioid trophoblastic tumor, or placental site nodule. Additionally, although the authors demonstrated increased vascularity on doppler ultrasonography, the color flow pattern was seen just at the uteroplacental contiguity, not all around the mass.

The presented case was a dichorionic diamniotic twin pregnancy in a nulliparous pregnant woman with 2 previous first trimester curettage operations. Authors said that the third stage of labor was complicated by retained placenta, and placentas were extracted manually and with banjo curettage under ultrasound guidance. It is possible that one of the placentas or a cotyledon was retained, was left in situ, and was not perceived during elimination process towards the placenta.

In our opinion, considering the suggested findings, this case is a typical presentation of a case of morbidly adherent placenta. It would be improper to build up a theory upon findings which does not meet the GTN criteria exactly, and it would be improper to make a preliminary diagnosis of GCC in this unique case.
